# What about immigration? The closed-population assumption in research on intergenerational income mobility

**DOI:** 10.3389/fsoc.2026.1799499

**Published:** 2026-07-13

**Authors:** Ernesto F. L. Amaral, Shih-Keng Yen, Arthur Sakamoto

**Affiliations:** 1 Department of Sociology, Texas A&M University, College Station, TX, United States; 2 Department of Social Welfare, National Chung Cheng University, Minxiong, Taiwan; 3 Department of Sociology, Hong Kong Baptist University, Hong Kong, Hong Kong SAR, China

**Keywords:** closed-population assumption, immigration, income mobility, intergenerational income elasticity, second generation

## Abstract

Research on intergenerational income mobility is highly informative and increasingly popular. A neglected aspect of these studies, however, is their implicit target population. Studies of income mobility are typically biased toward the representation of third-and-higher-generation persons. We refer to this practice as the closed-population assumption. First-generation, second-generation, and undocumented immigrants are less likely to be included because adequate data on income for their parental generation is more likely to be missing. Given significant international migration, ignoring the foreign stock is potentially inaccurate when making generalizations about entire societies. We discuss analytical issues associated with the closed-population assumption and provide some exploratory findings, which suggest significant interrelationships between emigration and intergenerational, income mobility.

## 1 Introduction

Research on intergenerational income mobility is highly informative and is becoming increasingly popular (Black and Devereux, 2011; Sakamoto and Wang, 2020). A neglected methodological issue regarding these studies, however, is that they are disproportionately based on individuals from later immigrant generations, particularly third-and-higher generation persons. In this study, we define immigrant generations as follows: First-generation individuals are those born outside the country of current residence; second-generation individuals are those born in the country of current residence whose parents were born outside the country of current residence; and third-and-higher-generation individuals are those born in the country of current residence whose parents were also born in the country of current residence. Accordingly, when we refer to “third-and-higher-generation persons,” we are referring to the third-and-higher generation of immigrants. The foreign stock (i.e., first-generation immigrants, including undocumented immigrants) and second-generation immigrants are often deleted or underrepresented in the samples used to estimate the intergenerational income elasticity (IGE). We refer to this approach as the closed-population assumption (i.e., deleting or underrepresenting the foreign stock and the second-generation of immigrants in research on intergenerational mobility), although this assumption is typically only implicit in most studies in this literature. Our empirical illustration is most clearly grounded in the U.S. context. However, the conceptual issues associated with the closed-population assumption apply more broadly across countries, with varying magnitude depending on migration regimes and data availability.

Recent research has emphasized that intergenerational mobility is shaped not only by family-level processes but also by broader structural, institutional, and spatial dynamics (Bailey et al., 2020; Bulczak and Gugushvili, 2022; Cheng and Wen, 2019; Movahed and Neman, 2024). This literature highlights how mobility processes are embedded in macro-level contexts, including inequality regimes, labor markets, and spatial opportunity structures. However, these approaches typically maintain the implicit closed-population assumption, focusing on individuals with complete intergenerational income data while overlooking how international migration may affect both the composition of observed populations and the interpretation of mobility estimates.

We discuss several analytical and measurement issues relating to the closed-population assumption. A recent study suggests that the insufficient inclusion of individuals from the 1.5- and second-generation may significantly affect the comprehension of disparities in cross-national income mobility (Sakamoto et al., 2023). At this stage of research, we offer several recommendations but we do not strongly argue in favor of any one particular methodological “solution” to account for the intergenerational mobility of immigrants. Our primary concern is to discuss why the closed-population assumption is important and to suggest avenues for future research. We also provide some exploratory results about the interrelationships between immigration and income mobility.

The objective of this study is to provide exploratory empirical evidence on the relationship between migration dynamics, particularly immigration and emigration, and cross-national variation in intergenerational income mobility. While the following sub-sections highlight the conceptual and methodological implications of the closed-population assumption, the empirical analysis focuses specifically on whether variation in migration patterns is associated with differences in observed IGE estimates across countries.

### 1.1 Data and measurement in prior studies

A defining methodological characteristic of both demographic analysis and inferential statistics is the analytical distinction between population and sample. The latter is used to make inferences about the characteristics of the former. This distinction may be considered trivial when population-level data is available. However, even in the limiting case of using population-level data, careful demographers will be concerned with the quality of the data in regard to missing values and the adequacy of population coverage in census data.

The IGE is commonly estimated based on Equation 1:


$$\begin{array}{l} log\left(Y_{offspring,i}\right)=\alpha +\beta log\left(Y_{parent,i}\right)+\varepsilon_{i\;} \end{array}$$ (1)

where *Y*_*offspring, i*_ is the adult income of offspring *i, Y*_*parent, i*_ is parental income, and β is the intergenerational income elasticity. A higher value of beta indicates a stronger association between parental and offspring income. Thus, higher IGE indicates greater intergenerational income persistence and lower intergenerational mobility. A lower value of beta indicates weaker persistence and higher intergenerational mobility. Because annual income contains transitory fluctuations, researchers often prefer income averaged across several years and measured at comparable life-course stages. If too few years of income are observed, transitory income measurement error may attenuate the estimated association between parental and offspring long-term income. Mazumder (2005) shows that even 5 years of income data may sometimes be insufficient, which means that IGE estimates may be biased downward (Black and Devereux, 2011) and intergenerational mobility may be overstated.

The underrepresentation of the foreign stock is common in studies of the IGE because such income data are often not available for first-generation and even second-generation immigrants. While population-level data may be generally adequate for vital statistics such as birth and death in contemporary developed nations, the income data needed to compute the IGE is far more extensive and typically less well known. As reviewed by Corak (2006), many studies of intergenerational mobility in the U.S. use data from the Panel Study of Income Dynamics (PSID). The target population for the PSID are American households in 1968, which excludes most post-1965 immigrants and their second-generation offspring (Corak, 2013). Because *approximately one in four Americans is first or second generation* in the contemporary U.S. (Trevelyan et al., 2016), conclusions based on the PSID do not apply to the entirety of the American population. An addition of several hundred new immigrant families were added to the PSID in 1997 (PSID Main Interview User Manual, 2019), but this sample is still too new to have a sufficient sample size of adult offspring to reliably estimate their long-term income.

Corak's (2006) review also indicates that many studies of intergenerational mobility use data from the 1979 National Longitudinal Survey (NLSY79). However, these data are based on a sample of persons born between 1957 and 1964, which is before the large increase in immigration to the U.S. after 1965. The sample for the 1997 National Longitudinal Survey (NLSY97) includes persons born between 1980 and 1984, so this cohort is beginning to mature enough that adult long-term income may be estimated soon (Mueller, 2014).

Another promising source of data to estimate the IGE are administrative records (Grusky et al., 2015). An influential study of this sort uses data from U.S. federal income tax returns from 1994 to 2015 matched to U.S. Census Bureau data from the 2000 Census, the 2010 Census, and the American Community Surveys from 2005 to 2015 (Chetty et al., 2020). The target population is all children born between 1978 and 1983 whose parents were legally residing in the U.S. and who were claimed as a child dependent on a federal income tax form in at least 1 year from 1994 to 2015. To be included in the sample, the child also needs to be linked to 5 years of the tax data for 1994–1995, and 1998–2000. Finally, the child's own income needs to be linked to W-2 forms for 2014 and 2015 when this cohort is between the ages of 31 and 37.

Chetty et al. (2020) is a path-breaking study, but it is worth nothing that several immigrant groups are still underrepresented by their methodology. The first is the obvious case of adult immigrants themselves who might be referred to as the 1.0-generation (Kim and Sakamoto, 2010). Their incomes in the U.S. may be substantially higher than the incomes of their parents when the 1.0-generation were still children in their countries of origin. The 1.0-generation often migrates to the U.S. for economic gain which is most prominent for immigrants coming from lower-income nations (e.g., Kaestner and Malamud, 2014). However, income gain is also a common theme even for high-skilled immigrants (Massey and Akresh, 2006). It is normally considered impossible to include the 1.0-generation into the calculation of the IGE because their parents are spread out around the world from a plethora of sending countries.

Nonetheless, from both an analytical as well as a policy point of view, the immigration of the 1.0-generation is still a substantively meaningful instance of income mobility. For example, if a country such as Finland has a lower rate of immigration of lesser-skilled persons compared to the U.S. (Belot and Hatton, 2012, p. 1111), then the comparison of the estimated IGE between these two countries (Corak, 2013, p. 82) might overestimate the greater income mobility in Finland compared to the IGE based on the complete residential populations.^1^ This cross-national comparison omits the greater income mobility in the U.S. in regard to having more lesser-skilled immigration associated with the 1.0-generation. Comparisons of Finland with other countries such as Canada or Germany could also be affected by differential patterns of the immigration of 1.0-generation.^2^

Relatedly, undocumented immigrants are not included in Chetty et al.'s (2020) target population. Their administrative records cannot be linked without a Social Security Number, which undocumented immigrants generally do not possess. This procedure also results in the native-born (i.e., second-generation) children of undocumented immigrants being excluded because their parental incomes are unavailable (Chetty et al., 2020, p. 725). Nations likely vary in the receipt of lower-skilled and other undocumented immigrants (Belot and Hatton, 2012). For example, if approximately 12 million undocumented immigrants resided in the U.S. in 2015 (Baker, 2018), then that would imply a higher rate of undocumented immigration to the U.S. than the rate of *all* immigration to some other countries in the Organisation for Economic Co-operation and Development (OECD) (Belot and Hatton, 2012).

Chetty et al. (2020) is based on data from many millions of administrative records. The large sample size yields informative results for the 1.5-generation [i.e., foreign-born persons who migrated to the U.S. at a young age (Kim and Sakamoto, 2010)]. However, the 1.5-generation is still underrepresented in their sample. Foreign-born children born in 1983 who did not arrive in the U.S. until 1995 or after would not be included due to a lack of sufficient years of data on parental income. Given continuous migration to the U.S. annually of various persons at all ages, some immigrant parents will inevitably be partially truncated from any available window of observation used to match administrative records.

Many immigrants study in graduate programs in the U.S. on student visas [who have also been referred to as the 1.25-generation (Kim and Sakamoto, 2010)] for several years. Their student visa status prevents them from legally working in the U.S., so they typically do not file any federal income tax returns. However, they often receive scholarships or other significant sources of income from their universities. Many of these student immigrants end up staying permanently in the U.S. (Bound et al., 2015) by way of H-1B and other visas. These immigrants may have foreign-born or native-born children. Those children (even when native born) may be less likely (in comparison to third-and-higher-generation persons) to be included in Chetty et al.'s (2020) target population due to the inadequate matching of parental income data in administrative records. Our concern is not to criticize Chetty et al.'s (2020) methodology *per se*, but simply to point out that the income mobility of first-generation and second-generation immigrants will inevitably be less likely to be matched in the records of administrative data.

Conclusions about changes across generations are influenced to a greater extent by the inadequate representation of individuals from the 1.5- and second-generation in the data utilized for estimating intergenerational income mobility (Sakamoto et al., 2023). This is particularly significant as the proportions of more recent cohorts expand over time within the population.

In sum, first-generation, second-generation and undocumented immigrants are typically underrepresented to varying degrees in the estimation of the IGE including in studies for countries other than the U.S. which employ similar methodologies (Corak, 2006). These groups are not always explicitly excluded in studies using longitudinal data, but the latter typically do not include sufficient information on the parental income of immigrants including those who arrived after the cohort being investigated.^3^ As mentioned earlier, this underrepresentation of the foreign stock in studies of intergenerational mobility may be referred to as the closed-population assumption.

Administrative data may be better able to consider more recent cohorts, but immigrants will invariably be less likely to be included in the sample due to less complete data on parental income or the partial truncation from the window of observation used to match administrative records. In both longitudinal surveys and administrative data, the income mobility of 1.0-generation immigrants is never even considered. This omission is presumably because collecting their parental income data would be nearly impossible even though their income mobility is theoretically relevant as well as pertinent to public policy debates. The focus on third-and-higher generation persons is not necessarily wrong from a purely technical point of view, but in these studies the closed-population assumption should at least be acknowledged.

### 1.2 Previous results about income mobility of the foreign stock

Chetty et al. (2020) is based on data from many millions of administrative records. Although likely undercounted as a percentage of the total sample, a sufficient sample size to estimate the IGE for the 1.5-generation is nonetheless available even after breaking down by racial groups including white people, Hispanics, African Americans, and Asians.^4^ For each of these 1.5-generation racial/ethnic groups, Chetty et al. (2020) find higher levels of upward intergenerational income mobility relative to their native-born counterparts.

Due to their higher levels of upward mobility, the underrepresentation of 1.5-generation immigrants from the total sample will lead to an upward bias (i.e., less mobility) in the estimation of the IGE for the U.S. as a whole. Studies of the second-generation in the U.S. suggest that these groups tend to have similarly higher levels of upward mobility; the second-generation likely does not differ greatly from the 1.5-generation (Farley and Alba, 2002; Glick and Hohmann-Marriott, 2007). The underrepresentation of second-generation groups from the total sample will therefore also lead to an upward bias in the estimation of the IGE for the U.S. as a whole.

However, countries likely vary regarding upward income mobility of the 1.5-generation and the second-generation. Compared to the U.S., opportunities for second-generation immigrants may be more limited in France (Simon, 2003; Algan et al., 2010), the Netherlands (Crul, 2000), Germany (Worbs, 2003; Schneider and Lang, 2014), and Denmark (Rytter, 2011). Obvious levels of second-generation disadvantage may be evident for some groups in various countries where second-generation persons are denied citizenship due to guest worker programs (Worbs, 2003; Fargues, 2011). On the other hand, higher levels of socioeconomic attainment often seem to be apparent in Canada, Australia, and the U.K. (Imoagene, 2012; Liu, 2014; Nguyen et al., 2020).

At this stage of research, strong conclusions about international differentials in second-generation income mobility might be premature for most OECD countries. Nonetheless, the current evidence does clearly suggest that such differentials are variable and relevant considerations. To the extent that international differentials in second-generation income mobility are significant, then the underrepresentation of second-generation immigrants from studies of the IGE could bias the results on cross-national comparisons.

### 1.3 Additional analytical issues

The basic theory of intergenerational income mobility is arguably implicitly based on the closed-population assumption by focusing on third-and-higher-generation persons while ignoring immigration. The IGE is described as referring to the extent to which the “permanent” (or “lifetime”) income of offspring is determined by or correlated with the “permanent” income of their parents (Black and Devereux, 2011). Income data from the middle-age range are generally preferred to estimate “permanent” income to avoid life-cycle bias, because income from the younger and older ages are generally believed to have a lower correlation with total “permanent” (or “lifetime”) income (Black and Devereux, 2011).

A potential analytical ambiguity in this approach is that, for first-generation immigrants, the parental income relevant to intergenerational mobility is usually the income their parents had in the country of origin when the immigrant was a child. The offspring's income, by contrast, is typically observed later in the country of destination. As noted above, this income differential is often precisely the primary motivation underlying immigration (Massey and Akresh, 2006). When an immigrant leaves a lower-income country and moves to a higher-income country, the income stream connecting parental resources in the origin country to offspring income in the destination country may be highly discontinuous. This discontinuity makes the standard “permanent income” framework more difficult to apply to first-generation immigrants than to third-and-higher-generation persons whose parents and offspring are observed within the same national income distribution.

For that reason, the “permanent income” conceptualization as the basis for the study of intergenerational mobility may be inappropriate. We suggest a reformulation that is based on the estimation of the IGE focused on the childhood income of the offspring rather than the “permanent income” or “lifetime income” of the parents. This approach has been formulated as the “linked lives” perspective developed by Cheng and Song (2019). They show that income flows at later years of the parental life course have a smaller association with offspring incomes than do parental income flows when the offspring was a child. Reformulating intergenerational mobility using childhood income as the focus would permit the analytical inclusion of immigrants into the conceptualization of the IGE.

This reformulation does not imply that offspring income should be measured at arbitrary ages. On the contrary, offspring income should still be measured at comparable adult ages to avoid life-cycle bias. The distinction is that parental income is theoretically most relevant when it captures resources available during the offspring's childhood, while offspring income is the adult outcome whose association with childhood resources is being estimated. From this perspective, the linked-lives approach requires attention to the timing of both generations, but the timing has different substantive meanings for parents and offspring.

Focusing on only the parental income during the time when the offspring was a child, the “linked lives” perspective seems compatible with micro-level models of intergenerational mobility. According to the Becker and Tomes (1979) model, the resources invested in children's social and educational investment are the key impact of parental income on the socioeconomic outcomes of offspring. Empirical support for this view has been presented by Heckman (2006) and Heckman and Mosso (2014), especially for investments made at younger ages of the child's development. Related conclusions about the importance of childhood socioeconomic resources for intergenerational mobility have long been made in various sociological studies (Sewell et al., 1969; Reardon, 2011; Sakamoto et al., 2014).

A second analytical issue to consider is the relevance of simulation methods to provide more informed estimates of the IGE for the full population in a given society. Akin to indirect standardization methods in demography, simulation analyses could use available information about the distribution of immigrant and native groups in a population, and then compute the expected overall statistical value, conditional upon reasonable proposed values on the group-specific rates.^5^ Obtaining complete parental income data may be infeasible for the 1.0-generation, but baseline values on their IGE could be reasonably estimated based on other information about skill levels and average incomes from principal sending countries by cohort. Estimates for income mobility for the 1.5-generation and the second-generation could be obtained from administrative records based on available data for those groups and then used to impute these observed IGE values for those portions of the 1.5-generation and the second-generation who are underrepresented in the observed sample. These simulation methods using census-level information on distribution of generational statuses could then provide useful baseline estimates of the IGE for the full population in a specific society.

A third analytical issue that we raise are the causal linkages between immigration and the IGE for the third-and-higher generation. By now, there is a large literature on the possible impacts of immigration on the incomes of native workers (e.g., Kim and Sakamoto, 2013; Borjas, 2014), which is beyond the scope of our discussion to summarize. Suffice to say here that even if analytically one's focus is on the IGE for the third-and-higher generation, understanding the casual sources of the latter may still require some consideration of immigration. The issue that has received much prior attention is whether the wages and other socioeconomic outcomes of native workers are reduced by the increase of immigrants with similar skills, which thereby increases the level of competition faced by native workers (Borjas et al., 2010). Conversely, immigrants may improve other sources of productivity and promote increased demand for native workers to upgrade their work skills (Hunt and Gauthier-Loiselle, 2010; Ottaviano and Peri, 2012; Card and Peri, 2016). The main conclusion from this literature is not that immigration has uniform effects on native-born workers. Rather, aggregate wage and employment effects are often limited, while effects may be more concentrated among workers who are closer substitutes for immigrant labor, including younger and less-educated native-born workers. For the present study, this implies that low-skilled immigration may not have a strong aggregate association with national IGE estimates, even if immigration affects subgroup-specific mobility patterns that cannot be evaluated with the country-level data used here. For our purposes, immigration may affect the IGE for the third-and-higher generation workers to the extent that their wages and employment are impacted.

However, what has been less well studied is the potential effect of emigration on the labor market outcomes of native workers (Aydemir and Borjas, 2007). If workers of a certain skill level exit a certain national labor market for employment in another country, then the supply of workers with that skill level will be reduced among the stayers in that national labor market. Wages and employment opportunities may thereby improve for workers who do not emigrate. Aydemir and Borjas (2007) find effects of this sort regarding emigration of different skill groups to Mexico, the U.S., and Canada. That is, in addition to the immigration of workers from other countries, patterns of emigration may affect the IGE for those third-and-higher generation workers who remain in their country.

The implications of emigration also depend on the age and skill composition of those leaving. Youth emigration may reduce the domestic population before adult earnings are observed, which can affect both the population denominator and the observed income distribution of stayers. Skilled emigration may remove individuals with high potential earnings, which can change the composition of the remaining labor force and the interpretation of national mobility estimates. Old-age or retirement migration is less directly connected to working-age IGE estimates, but it may still affect population registers and cross-national comparability if migration status is recorded differently across countries. Because the available cross-national data do not distinguish these mechanisms in detail, our empirical analysis treats emigration as an aggregate contextual measure rather than as a direct individual-level mechanism.

Overall, the preceding sub-sections provide the conceptual and methodological background motivating this study. Building on these considerations, we now turn to an exploratory empirical analysis of the relationship between migration dynamics and intergenerational income mobility.

## 2 Methods

We provide exploratory findings about the association between migration dynamics and the IGE for third-and-higher-generation persons. To our knowledge, our analysis is the first to investigate the relationship between IGE estimates and country-level measures of immigration and emigration. The unit of analysis is not an individual person, but a published estimate of intergenerational income elasticity for a given country and population. The *dependent variable* is the estimated IGE reported in prior published research and compiled by Amaral et al. (2019). All IGE estimates included in our models refer to years after 2001. Throughout the analysis, we use the definitions of immigrant generations introduced above, with “third-and-higher-generation individuals” referring to persons born in the country of current residence whose parents were also born in that country.

Standard estimates of the IGE primarily reflect the experiences of individuals with long-standing family residence in the country of observation. Because intergenerational income data are more readily available for these populations, IGE estimates disproportionately capture the mobility patterns of “stayers” and third-and-higher-generation persons, rather than the full resident population inclusive of recent immigrants and their descendants.

The *independent variables* are country-level migration measures from the Database on Immigrants in OECD and non-OECD Countries (DIOC) for reference years 2000 to 2001 (https://www.oecd.org/els/mig/dioc.htm). We use three measures. The first is the proportion of immigrants with less than secondary schooling. For this measure, country *j* is treated as the destination country, and the numerator is the number of foreign-born residents with less than secondary schooling. The second is the proportion of emigrants. For this measure, country *j* is treated as the origin country, and the numerator is the number of persons from country *j* residing abroad. The third is the proportion of emigrants with tertiary education, which restricts the emigration numerator to tertiary-educated emigrants from country *j*. In our analysis, each measure is expressed relative to the resident population of country *j*.

The emigration variables capture aggregate country-level population exposure. They do not distinguish return moves of previous immigrants, onward migration, native-born emigrants by immigrant generation, age at departure, or timing of emigration. For this reason, emigration is interpreted as a country-level contextual measure rather than as a direct individual-level mechanism.

All variables used in this study are derived from publicly available sources, and replication is possible using the data and procedures described in Amaral et al. (2019) and the OECD DIOC database (2000–2001). The temporal structure of the variables follows a conceptually appropriate ordering. The migration measures (proportions of immigrants and emigrants) are based on reference years 2000–2001 and are treated as prior population characteristics, while the IGE estimates are derived from cohorts observed in subsequent years. This sequencing allows migration dynamics to be interpreted as preceding the observed mobility outcomes, thereby avoiding concerns of reverse or simultaneous relationships.

We estimate two ordinary least squares regression models, as illustrated in Equations 2, 3:


$$\begin{array}{l} \text{IG}{\text{E}}_{\text{jp}}=\alpha +\beta_{1}\text{ImmigPrimar}{\text{y}}_{\text{j}}+\beta_{2}\text{Emi}{\text{g}}_{\text{j}}+\delta_{\text{p}}+\varepsilon_{\text{jp}} \end{array}$$ (2)

$$\begin{array}{l} \text{IG}{\text{E}}_{\text{jp}}=\alpha +\beta_{1}\text{ImmigPrimar}{\text{y}}_{\text{j}}+\beta_{2}\text{EmigTertiar}{\text{y}}_{\text{j}}+\delta_{\text{p}}+\varepsilon_{\text{jp}} \end{array}$$ (3)

where *IGE*_*jp*_ is the intergenerational income elasticity estimate for country *j* from source publication *p*, ImmigPrimary_*j*_ is the proportion of the resident population composed of immigrants with less than secondary schooling, Emig_*j*_ is the proportion of emigrants, EmigTertiary_*j*_ is the proportion of emigrants with tertiary education, and δ_*p*_ represents publication fixed effects.

Higher values of IGE indicate greater similarity between parental income and offspring income, which implies lower levels of intergenerational mobility. Lower values of IGE indicate weaker intergenerational income persistence, which implies higher levels of intergenerational mobility. Thus, in our regression models, statistically significant negative coefficients indicate that higher values of a migration measure are associated with lower observed IGE values and therefore higher observed intergenerational mobility. Because the analysis is cross-national and exploratory, these coefficients should be interpreted as aggregate associations rather than causal effects.

The models include fixed effects for source publication, not country fixed effects. Country fixed effects are not feasible because the migration measures are defined at the country level for 2000–2001 and therefore do not vary within country. Including country fixed effects would absorb the main explanatory variables. Publication fixed effects instead adjust for systematic differences across source studies, including differences in data sources, measurement strategies, sample definitions, and estimation procedures. Standard errors are clustered by source publication to allow intragroup correlation across IGE estimates drawn from the same study.^6^

To make the analysis replicable, the construction of the analytic dataset proceeds in four steps. First, we extract country-specific IGE estimates reported in Amaral et al. (2019). Second, we retain estimates whose observed income period occurs after 2001. Third, we attach DIOC 2000 to 2001 country-level migration variables to each country represented in the IGE dataset. Fourth, we estimate OLS models with publication fixed effects and standard errors clustered by source publication. The replication data include the country, source publication, IGE estimate, DIOC migration variables, and the publication identifier used for fixed effects and clustering.

As shown in Table 1, our investigation includes data for 20 different countries gleaned from previous studies in the ever-expanding literature on the IGE. For most (but not all) of the countries, multiple estimates of the IGE are available. Including each of the different estimates based on slightly varying data sets, demographic groups, and methods, our total sample size is 130 as is reported in Table 1. For example, Table 1 shows that our data set includes nine estimates for Spain, four estimates for Sweden, one estimate for Switzerland, 13 estimates for the United Kingdom, and 25 estimates for the U.S.

**Table 1:** Number of intergenerational income elasticity (IGE) observations by country.

Countries	Frequency	Percent
Australia	12	9.23
Brazil	2	1.54
Canada	21	16.15
Chile	1	0.77
Denmark	18	13.85
Finland	4	3.08
France	3	2.31
Germany	4	3.08
Italy	3	2.31
Japan	1	0.77
New Zealand	1	0.77
Norway	4	3.08
Peru	1	0.77
Singapore	1	0.77
South Africa	2	1.54
Spain	9	6.92
Sweden	4	3.08
Switzerland	1	0.77
United Kingdom	13	10.00
United States	25	19.23
Total	130	100.00

Database generated by the authors with: (1) measure of intergenerational income elasticity from a series of previously published studies (Amaral et al., 2019); and (2) proportion of immigrants and proportion of emigrants from the Database on Immigrants in OECD and non-OECD Countries (DIOC) for reference years 2000–2001 (https://www.oecd.org/els/mig/dioc.htm).

## 3 Results

Table 2 shows the results for the regression models which are estimated by ordinary least squares. In Model 1, the independent variables include the proportion of immigrants who have less than a secondary schooling degree, and the proportion of emigrants. In Model 2, the independent variables include the proportion of immigrants who have less than a secondary schooling degree, and the proportion of emigrants who have a tertiary schooling degree.

**Table 2:** Estimates from ordinary least squares (OLS) regression models of intergenerational income elasticity (IGE) on migration measures.

Independent variables	Model 1	Model 1 (standardized coefficient)	Model 2	Model 2 (standardized coefficient)
Constant	0.379^**^ (0.023)		0.356^**^ (0.023)	
Proportion of immigrants (primary educated)	0.036	0.027	0.067	0.050
	(0.174)		(0.171)	
Proportion of emigrants	−1.847^**^ (0.522)	−0.323	
Proportion of emigrants (tertiary educated)			−1.014^*^ (0.464)	−0.265
Fixed effects	Yes	Yes	Yes	Yes
*R* ^2^	0.454		0.434	
Adjusted *R*^2^	0.336		0.311	
Observations	130		130	

Intergenerational income elasticity (IGE) is the dependent variable in all models. Model 1 includes the proportion of immigrants (primary educated) and the proportion of emigrants as independent variables. Model 2 includes the proportion of immigrants (primary educated) and the proportion of emigrants (tertiary educated). Standardized coefficients are reported in adjacent columns for each model. All models include fixed effects for publication. Fixed-effects are not country-specific but rather refer to the publication that provided the value of the dependent variable. Robust standard errors are reported in parentheses, which allow for intragroup correlation within publications. Replication data include the country, source publication, IGE estimate, DIOC migration variables, and publication identifier used for fixed effects and clustered standard errors.

^**^Significant at *p* < 0.01. ^*^Significant at *p* < 0.05.

Intergenerational income elasticity is from a series of previously published studies (Amaral et al., 2019). Proportion of immigrants and proportion of emigrants are from the Database on Immigrants in OECD and non-OECD Countries (DIOC) for reference years 2000–2001 (https://www.oecd.org/els/mig/dioc.htm).

The results in Table 2 for Model 1 indicate that the coefficient for less-educated immigrants is small and not statistically significant. Net of emigration, no statistically significant aggregate association between low-skilled immigration and IGE is apparent for third-and-higher-generation persons. However, Table 2 also shows that the coefficient for the proportion of emigrants is highly statistically significant in Model 1. The coefficient of −1.847 indicates that an increase of 0.1 in the proportion of persons who leave a particular country is associated with a lower IGE by −0.1847, on average. The standardized effect for this result is −0.323, which may be interpreted as a one-standard-deviation unit increase in emigration reducing the IGE by 0.323 of a standard-deviation unit. This finding means that higher emigration is associated with higher observed levels of intergenerational mobility, as indicated by smaller IGE values.

In Model 2 of Table 2, the coefficient for less-educated immigrants is again substantively small and not statistically significant. Net of emigration, no statistically significant aggregate association between low-skilled immigration and IGE is discernible in Model 2. In this case, the emigration variable is restricted to the proportion who have a tertiary degree. Table 2 shows that the coefficient for highly-educated emigrants is statistically significant in Model 2. The coefficient of −1.014 indicates that an increase of 0.1 in the proportion of persons who leave a particular country is associated with a lower IGE by −0.1014, on average. The standardized effect for this result is −0.265. This effect of highly-educated emigration is generally substantial, but not as large as the estimate for all emigration as obtained in Model 1.

## 4 Discussion

Besides Sakamoto et al. (2023), we are unaware of other studies of the IGE that even mentions immigration as a substantive issue (i.e., except perhaps to briefly note that immigrants are deleted from sample). Our primary objective in this paper is simply to raise awareness of the theoretical and methodological implications of this issue which we refer to as the closed-population assumption. Ignoring the foreign stock seems unrealistic for the purpose of describing the level of intergenerational mobility in a nation with a significant proportion of immigrants. In a world characterized by rising international migration that is often motivated by economic mobility, the closed-population assumption yields conclusions that might not accurately represent the entire society in question.

When making cross-national comparisons, disregarding the income mobility associated with foreign-born immigrants leads to potentially myopic conclusions because countries vary dramatically in their openness to immigration. For example, approximately one in three persons in Australia is foreign-born while about one in a 100 persons in Japan is foreign-born (Belot and Hatton, 2012). Income mobility generated by immigration is therefore probably far greater in Australia than in Japan. Statistics describing the IGE among the third-and-higher generation are not wrong *per se* if the latter group is the explicit focus of the analysis. However, researchers generalizing about societies as a whole population (e.g., Australia vs. Japan) need to explicitly state their closed-population assumption, as well as bear in mind that immigrants are a component of the resident population in every modern society.

In terms of understanding the sources of IGE for third-and-higher-generation persons, our exploratory findings suggest that emigration deserves further attention. A larger proportion of persons leaving a country is associated with lower observed IGE, which implies higher observed intergenerational mobility. This association should be interpreted cautiously. It is consistent with the possibility that emigration changes the composition of the remaining labor force or improves opportunities for stayers, but the cross-national design cannot rule out confounding from institutions, registration practices, or broader economic conditions. Future research should investigate this topic with harmonized longitudinal data that can distinguish age at emigration, skill level, return migration, onward migration, and immigrant generation.

While obtaining complete income data for all the components of the first-generation and second-generation seems unlikely, simulation methods might be employed to provide more informed estimates of the IGE for the full population in a given society. Simulation analyses could use available information about the distribution of immigrant and native groups in a population. This data could be used to compute the expected overall statistical effects of immigration on intergenerational mobility, conditional upon reasonable proposed values (based on other available information) on the group-specific rates. These simulated IGE values for the full population in a particular society could then be compared to the IGE values based on only the third-and-higher generation, which is similar to indirect standardization in demography.

While the theoretical basis of the IGE has been traditionally based on the idea of “permanent income,” this approach might be reformulated to incorporate the “linked lives” perspective, which can accommodate both the third-and-higher generation and the foreign stock. We argue for further research on this topic because underrepresenting immigrants is increasingly unrealistic for the purposes of describing the level of intergenerational income mobility in contemporary societies. In a world characterized by rising international migration, which is often motivated by economic opportunities for income mobility, the closed-population assumption is yielding cross-national comparisons that are limited and potentially misleading.

This study has several limitations. First, the empirical analysis is descriptive and illustrative rather than causal, and the results should not be interpreted as identifying structural determinants of intergenerational mobility. Second, the models do not include institutional or policy variables (e.g., welfare regimes or education systems), which may also shape mobility outcomes. Third, there is a temporal mismatch between migration measures (2000–2001) and IGE estimates derived from multiple cohorts and years. This approach preserves the correct temporal ordering, where migration dynamics precede observed mobility outcomes. However, it may still introduce some measurement noise. Fourth, the cross-national design does not support within-country estimation. The migration variables are country-level measures for 2000 to 2001 and therefore do not vary within countries. Multiple observations per country refer to different published IGE estimates, not repeated harmonized country-year observations. Fifth, emigration measures do not distinguish return migration, onward migration, age at departure, immigrant generation, or the timing of emigration. These limitations mean that the emigration results should be interpreted as exploratory aggregate associations rather than as evidence of a direct individual-level mechanism. Sixth, cross-national differences in the definition and measurement of “immigrant” may affect comparability across countries. Finally, standard IGE measures themselves are subject to data limitations, particularly regarding the underrepresentation of immigrant populations.

## Data Availability

The original contributions presented in the study are included in the article/supplementary material, further inquiries can be directed to the corresponding author.

## Footnotes

1 Immigration can also affect welfare implications when making international comparisons of poverty rates (Sasson and Sakamoto, 2014).

2 According to (Corak 2013, p. 82), the IGE is low in Finland, high in the U.S., and intermediate in Germany and Canada.

3 As stated by (Jäntti and Jenkins 2015, p. 860), “If a country experiences significant immigration, a panel inevitably becomes unrepresentative of the population in later years. Sample refreshment has been employed to counter this problem, but if mobility estimates are required for time points spanning the old and new population structures, refreshment cannot improve representativeness.”

4 Unfortunately, Chetty et al. (2020) do not report their sample sizes broken down by generational status.

5 For a generic example of this sort, see Breen and Chung's (2015) simulation analysis of the relationships between educational attainment and income distribution.

6 The standard errors are not based on the conventional OLS formula, however, because they are robust estimates that also allow for intragroup correlation among values of the dependent variable that are obtained from the same publication.

## References

[B1] Algan Y. Dustmann C. Glitz A. Manning A. (2010). The economic situation of first and second-generation immigrants in France, Germany and the United Kingdom. Econ. J. 120, 4–30. doi: 10.1111/j.1468-0297.2009.02338.x

[B2] Amaral E. F. L. Yen S. K. Wang-Goodman S. X. (2019). A meta-analysis of the association between income inequality and intergenerational mobility. Socius Sociol. Res. Dyn. World 5, 1–18. doi: 10.1177/2378023119881282

[B3] Aydemir A. Borjas G. J. (2007). Cross-country variation in the impact of international migration: Canada, Mexico, and the United States. J. Eur. Econ. Assoc. 5, 663–708. doi: 10.1162/JEEA.2007.5.4.663

[B4] Bailey M. J. Cole C. Henderson M. Massey C. (2020). How well do automated linking methods perform? Lessons from US historical data. J. Econ. Lit. 58, 997–1044. doi: 10.1257/jel.20191526 34294947 PMC8294155

[B5] Baker B. (2018). Estimates of the Illegal Alien Population Residing in the United States: January 2015. Department of Homeland Security, Office of Immigration Statistics. Washington, D.C.

[B6] Becker G. S. Tomes N. (1979). An equilibrium theory of the distribution of income and intergenerational mobility. J. Polit. Econ. 87, 1153–1189. doi: 10.1086/260831

[B7] Belot M. V. Hatton T. J. (2012). Immigrant selection in the OECD. Scand. J. Econ. 114, 1105–1128. doi: 10.1111/j.1467-9442.2012.01721.x

[B8] Black S. Devereux P. J. (2011). “Recent developments in intergenerational mobility,” in Handbook of Labor Economics, Vol. 4B, eds. O. Ashenfelter, and D. Card (New York: Elsevier), 1487–1541. doi: 10.1016/S0169-7218(11)02414-2

[B9] Borjas G. J. (2014). Immigration Economics. Cambridge: Harvard University Press. doi: 10.4159/harvard.9780674369900

[B10] Borjas G. J. Grogger J. Hanson G. H. (2010). Immigration and the economic status of African-American men. Economica 77, 255–282. doi: 10.1111/j.1468-0335.2009.00803.x

[B11] Bound J. Demirci M. Khanna G. Turner S. (2015). Finishing degrees and finding jobs: US higher education and the flow of foreign IT workers. Innov. Policy Econ. 15, 27–72. doi: 10.1086/680059

[B12] Breen R. Chung I. (2015). Income inequality and education. Sociol. Sci. 2, 454–477. doi: 10.15195/v2.a22

[B13] Bulczak G. Gugushvili A. (2022). Downward income mobility among individuals with poor initial health is linked with higher cardiometabolic risk. PNAS Nexus 1, 1–9. doi: 10.1093/pnasnexus/pgac012 36712801 PMC9802411

[B14] Card D. Peri G. (2016). Immigration economics by George J. Borjas: a review essay. J. Econ. Lit. 54, 1333–1349. doi: 10.1257/jel.20151248

[B15] Cheng S. Song X. (2019). Linked lives, linked trajectories: intergenerational association of intragenerational income mobility. Am. Sociol. Rev. 84, 1037–1068. doi: 10.1177/0003122419884497

[B16] Cheng S. Wen F. (2019). Americans overestimate the intergenerational persistence in income ranks. PNAS 116, 13909–13914. doi: 10.1073/pnas.1814688116 31235566 PMC6628797

[B17] Chetty R. Hendren N. Jones M. R. Porter S. R. (2020). Race and economic opportunity in the United States: an intergenerational perspective. Q. J. Econ. 135, 711–783. doi: 10.1093/qje/qjz042

[B18] Corak M. (2006). Do poor children become poor adults? Lessons from a cross-country comparison of generational earnings mobility. Res. Econ. Inequal. 13, 143–188. doi: 10.1016/S1049-2585(06)13006-9

[B19] Corak M. (2013). Income inequality, equality of opportunity, and intergenerational mobility. J. Econ. Perspect. 27, 79–102. doi: 10.1257/jep.27.3.79

[B20] Crul M. (2000). “Breaking the circle of disadvantage. Social mobility of second-generation Moroccans and Turks in the Netherlands,” in Immigrants, Schooling and Social Mobility (London: Palgrave Macmillan), 225–244. doi: 10.1057/9780333985502_11

[B21] Fargues P. (2011). Immigration without inclusion: non-nationals in nation-building in the Gulf states. Asian Pac. Migr. J. 20, 273–292. doi: 10.1177/011719681102000302

[B22] Farley R. Alba R. (2002). The new second generation in the United States. Int. Migr. Rev. 36, 669–701. doi: 10.1111/j.1747-7379.2002.tb00100.x

[B23] Glick J. E. Hohmann-Marriott B. (2007). Academic performance of young children in immigrant families: the significance of race, ethnicity, and national origins. Int. Migr. Rev. 41, 371–402. doi: 10.1111/j.1747-7379.2007.00072.x

[B24] Grusky D. B. Smeeding T. M. Snipp C. M. (2015). A new infrastructure for monitoring social mobility in the United States. Ann. Am. Acad. Pol. Soc. Sci. 657, 63–82. doi: 10.1177/0002716214549941 30111895 PMC6089542

[B25] Heckman J. J. (2006). Skill formation and the economics of investing in disadvantaged children. Science 312, 1900–1902. doi: 10.1126/science.1128898 16809525

[B26] Heckman J. J. Mosso S. (2014). The economics of human development and social mobility. Annu. Rev. Econom. 6, 689–733. doi: 10.1146/annurev-economics-080213-040753 25346785 PMC4204337

[B27] Hunt J. Gauthier-Loiselle M. (2010). How much does immigration boost innovation? Am. Econ. J. Macroecon. 2, 31–56. doi: 10.1257/mac.2.2.31

[B28] Imoagene O. (2012). Being British vs being American: identification among second-generation adults of Nigerian descent in the US and UK. Ethn. Racial Stud. 35, 2153–2173. doi: 10.1080/01419870.2011.631556

[B29] Jäntti M. Jenkins S. P. (2015). “Income mobility,” in Handbook of Income Distribution, Vol. 2. (Amsterdam: Elsevier), 807–935. doi: 10.1016/B978-0-444-59428-0.00011-4

[B30] Kaestner R. Malamud O. (2014). Self-selection and international migration: new evidence from Mexico. Rev. Econ. Stat. 96, 78–91. doi: 10.1162/REST_a_00375

[B31] Kim C. Sakamoto A. (2010). Have Asian American men achieved labor market parity with white men?. Am. Sociol. Rev. 75, 934–957. doi: 10.1177/0003122410388501

[B32] Kim C. Sakamoto A. (2013). Immigration and the wages of native workers: spatial versus occupational approaches. Sociol. Focus 46, 85–105. doi: 10.1080/00380237.2013.766834

[B33] Liu X. (2014). Educational attainment of second-generation immigrants: a US-Canada comparison. IZA Discussion Papers, No. 8685, Institute for the Study of Labor (IZA), Bonn, Germany. doi: 10.2139/ssrn.2534713

[B34] Massey D. S. Akresh I. R. (2006). Immigrant intentions and mobility in a global economy: the attitudes and behavior of recently arrived US immigrants. Soc. Sci. Q. 87, 954–971. doi: 10.1111/j.1540-6237.2006.00410.x

[B35] Mazumder B. (2005). The apple falls even closer to the tree than we thought. Unequal Chances Fam. Background Econ. Success 80–99. doi: 10.2307/j.ctt7tbdz.6

[B36] Movahed M. Neman T. (2024). Intergenerational income mobility in the United States: a racial-spatial account. Soc. Sci. Res. 123:103064. doi: 10.1016/j.ssresearch.2024.103064 39256025

[B37] Mueller C. (2014). The intergenerational income elasticity in the NLSY97 dataset. Graduate Report No. 396, Utah State University. Available online at: https://digitalcommons.usu.edu/cgi/viewcontent.cgi?article=1400andcontext=gradreports (Accessed January 15, 2026).

[B38] Nguyen H. T. Connelly L. B. Le H. T. Mitrou F. Taylor C. L. Zubrick S. R. (2020). Ethnicity differentials in academic achievements: the role of time investments. J. Popul. Econ. 33, 1381–1418. doi: 10.1007/s00148-020-00774-6

[B39] Ottaviano G. I. Peri G. (2012). Rethinking the effect of immigration on wages. J. Eur. Econ. Assoc. 10, 152–197. doi: 10.1111/j.1542-4774.2011.01052.x

[B40] PSID Main Interview User Manual (2019). Institute for Social Research, University of Michigan, February, 2019. Available online at: https://psidonline.isr.umich.edu/data/Documentation/UserGuide2017.pdf (Accessed January 15, 2026).

[B41] Reardon S. F. (2011). “The widening academic achievement gap between the rich and the poor: new evidence and possible explanations”, in Whither Opportunity?, eds. G. J. Duncan and R. J. Murnane, Vol. 1, 91–116. Available online at: https://www.russellsage.org/publications/whither-opportunity (Accessed January 15, 2026).

[B42] Rytter M. (2011). Money or education? Improvement strategies among Pakistani families in Denmark. J. Ethn. Migr. Stud. 37, 197–215. doi: 10.1080/1369183X.2011.521356

[B43] Sakamoto A. Bloome D. Amaral E. F. L. Daumler D. Yen S. K. (2023). Open-population inference: an investigation of the underrepresentation of the second generation in research on intergenerational income mobility. Int. Migr. Rev. 57, 1249–1278. doi: 10.1177/01979183221126462

[B44] Sakamoto A. Rarick J. Woo H. Wang S. X. (2014). What underlies the Great Gatsby Curve? Psychological micro-foundations of the “vicious circle” of poverty. Mind Soc. 13, 195–211. doi: 10.1007/s11299-014-0144-x

[B45] Sakamoto A. Wang S. X. (2020). The declining significance of occupation in research on intergenerational mobility. Res. Soc. Stratif. Mobil. 70:100521. doi: 10.1016/j.rssm.2020.100521

[B46] Sasson I. Sakamoto A. (2014). Non-poor components of population growth and immigration in the US, 1990–2010. Soc. Indic. Res. 115, 183–201. doi: 10.1007/s11205-012-0214-6

[B47] Schneider J. Lang C. (2014). Social mobility, habitus and identity formation in the Turkish-German second generation. New Divers. 16, 89–105.

[B48] Sewell W. H. Haller A. O. Portes A. (1969). The educational and early occupational attainment process. Am. Sociol. Rev. 34, 82–92. doi: 10.2307/2092789

[B49] Simon P. (2003). France and the unknown second generation: preliminary results on social mobility. Int. Migr. Rev. 37, 1091–1119. doi: 10.1111/j.1747-7379.2003.tb00171.x

[B50] Trevelyan E. N. Gambino C. Gryn T. Larsen L. Acosta Y. Grieco E. M. . (2016). Characteristics of the US Population by Generational Status, 2013. US Department of Commerce, Economic and Statistics Administration, US Census Bureau.

[B51] Worbs S. (2003). The second generation in Germany: between school and labor market. Int. Migr. Rev. 37, 1011–1038. doi: 10.1111/j.1747-7379.2003.tb00168.x

